# Enhanced Anaerobic Biogas Production From Wheat Straw by Herbal-Extraction Process Residues Supplementation

**DOI:** 10.3389/fbioe.2021.623594

**Published:** 2021-05-06

**Authors:** Yonglan Xi, Yang Liu, Xiaomei Ye, Jing Du, Xiangping Kong, Dong Guo, Qingbo Xiao

**Affiliations:** ^1^East China Scientific Observing and Experimental Station of Development and Utilization of Rural Renewable Energy, Ministry of Agriculture, Nanjing, China; ^2^Laboratory for Agricultural Wastes Treatment and Recycling, Recycling Agriculture Research Center, Jiangsu Academy of Agricultural Sciences, Nanjing, China; ^3^School of Agricultural Equipment Engineering, Jiangsu University, Zhenjiang, China

**Keywords:** wheat straw, Chinese herbal medicine residues, anaerobic digestion, biogas, methane

## Abstract

Trace metals are essential constituents of cofactors and enzymes and that their addition to anaerobic digesters increases methane production. Many trace elements are contained in herbal-extraction process residues (HPR). The present study concerns the effect of six kinds of HPR [Danshen root (Dr), Astragalus membranaceus (Am), Isatis root (Ir), *Angelica sinensis* (As), and Pseudo-ginseng (Pg)] that were used as additives, respectively, in the anaerobic digestion of wheat straw on biogas and methane production. The ratios of HPR residues/wheat straw [based on total solids (TS), of wheat straw] were 3, 5, and 10%, respectively. The digesters were at 37 ± 1°C of water bath during 30 days of anaerobic digestion. The results showed that HPR had significant effects on the anaerobic co-digestion. The highest biogas productivity was achieved when treated with 10% Pseudo-ginseng residues (PGR), which yielded 337 ml/g TS of biogas and 178 ml/g TS of methane. Cumulative production of biogas and methane increased by 28 and 37% compared to the production achieved in the control. These results suggest that PGR is an effective HPR to enhance the production of methane.

## Introduction

Methane production through anaerobic digestion of biomass has been considered as an attractive and potential method to obtain renewable biofuel due to its great environmental, societal, and economic benefits (Zhang et al., 2014; Rasapoor et al., 2020). Anaerobic digestion (AD) is a naturally occurring phenomenon in which organic matter is decomposed by an assortment of microbes in an oxygen-free environment to produce biogas, composed primarily of methane (CH_4_) and carbon dioxide (CO_2_) (Frigon and Guiot, 2010; Esposito et al., 2012; Kumar and Samadder, 2020). China is a major agricultural country rich in the resources of biomass. Wheat straw as agricultural residues is considered an abundant renewable resource. The total annual production of wheat straw in China was 109 million metric tons in 2007 (Yang et al., 2010). The anaerobic digestion used in dealing with wheat straw is an attractive practice in which both pollution control and energy recovery can be achieved (Solé-Bund et al., 2017).

Meanwhile, anaerobic digestion is a sensitive multi-stage process (hydrolysis, acidogenesis, acetogenesis, and methanogenesis) that depends on susceptible microorganisms to carry out the digestion job (Li et al., 2014; Mata-Alvarez et al., 2014). The growth and passage of microorganisms of AD require the involvement of many trace elements (Zhang et al., 2019). One of the influential factors is the presence of trace elements in the digestion system which mainly function as micronutrients. The trace elements must be adequate for supporting the metabolism of the microorganisms to maintain the effective digestion process (Choong et al., 2016; Wang et al., 2017). However, the solid organic substrates such as energy crops, crop residues (wheat straw included), and organic fraction of municipal solid waste (OFMSW) have the natural characteristics of the low trace element content. This aspect has been highlighted by researchers and many studies have been carried out to demonstrate the importance of trace elements in anaerobic digestion. Yu has reported that ferric chloride (FeCl_3_), as an additive, was supplemented into the sludge thermophilic AD system has directly enhanced methane production (Yu et al., 2016). The methane production was improved by 7–15% when Ni/Mo/B and Se/W were added in the digesters (Feng et al., 2009). Harris reported that raw Chinese herbal medicine contained an abundance of trace elements (Harris et al., 2011). HPR, as a biomass resource, is easy to decay and is potentially harmful to the environment (Cheng and Liu, 2010; Wang et al., 2010). With the rapid development of the Chinese herbal medicine industry, over 10 million tons of HPR per year are produced (Wu et al., 1998). How to reuse and recycle this valuable biomass resource is a very urgent job (Zhou et al., 2014, 2016). Therefore, it is a better option to reuse HPR as the function of trace elements in the anaerobic digestion (Rasdi et al., 2013; Xi et al., 2014).

The objective of this study was to investigate HPR as an additive for enhancing methane production from the anaerobic digestion of wheat straw. In this way, it does not only help resolve the low trace element in the digesters but is also a good option to reuse HPR. We have selected Danshen root (Dr), Astragalus membranaceus (Am), Isatis root (Ir), *Angelica sinensis* (As), Pseudo-ginseng (Pg), and *Codonopsis pilosula* (Cp) residues to detect their physicochemical property and add them in the AD system at an appropriate ratio. They were investigated during a 30-d anaerobic digestion period in batch anaerobic reactors operated under mesophilic conditions. To our knowledge, this is the first study to identify the specific type of Chinese medicinal residues to enhance methane production from the anaerobic digestion of wheat straw.

## Experimental

### Substrates and Inoculums

Wheat straw was freshly collected from a farmyard in Jiangsu Academy of Agricultural Science, Nanjing, Jiangsu Province, China at the end of May 2016. Wheat straw was first air-dried then was crushed to the size of ~1 mm using a grinder (Hummer 900, USA). Chinese herbal medicine was purchased from Nanjing Jinling drug store (Nanjing, China) and then kept in boiling water for 20 min. After that, the filter removed the soup. HPR was dried in the oven at 80°C for 6 h. Finally, the particles were cut to the size of ~1 mm using a grinder and stored at 4°C until use. Table 1 shows the chemical parameters of wheat straw and HPR.

**Table 1:** Characteristics of wheat straw, HPR, and anaerobic sludge.

**Parameter**	**Wheat straw**	**Am** ** residues**	**Ds** ** residues**	**Ir** ** residues**	**Pg** ** residues**	**Cp residues**	**As** ** residues**	**Anaerobic sludge**
Total solids (Ts/%)	89.74 ± 0.02^a^	95.06 ± 0.05	89.63 ± 0.02	95.65 ± 0.03	91.63 ± 0.05	91.29 ± 0.01	93.91 ± 0.02	3.21 ± 0.04
Volatile solids (Vs/%)	87.91 ± 0.02	97.82 ± 0.06	94.41 ± 0.04	97.20 ± 0.03	97.78 ± 0.05	93.53 ± 0.02	93.75 ± 0.02	64.37 ± 2.12
Total carbon (/%)	33.53 ± 0.37	39.55 ± 0.50	15.48 ± 0.06	39.55 ± 0.32	49.01 ± 0.50	41.27 ± 0.53	43.85 ± 0.05	486.33 ± 12.26
Total nitrogen (/%)	0.35 ± 0.02	1.74 ± 0.15	3.27 ± 0.43	2.13 ± 0.32	0.78 ± 0.03	1.32 ± 0.22	2.48 ± 0.15	10.52 ± 0.16
C/N	95.00	22.78	4.73	18.59	62.51	31.20	17.70	46.23
Cellulose (/%)	34.02 ± 0.03	32.06 ± 0.41	30.24 ± 0.35	6.23 ± 0.41	5.20 ± 0.06	8.92 ± 0.13	6.67 ± 0.06	na^c^
Hemicelluloses (/%)	27.58 ± 0.22	22.22 ± 0.13	22.97 ± 0.02	45.44 ± 0.12	39.17 ± 0.24	9.46 ± 0.05	8.53 ± 0.15	na
Lignin (/%)	17.48 ± 0.23	4.54 ± 0.03	13.38 ± 0.08	2.65 ± 0.10	0.44 ± 0.34	2.11 ± 0.01	2.50 ± 0.06	na
Starch (/%)	0.43 ± 0.04	8.05 ± 0.06	0.37 ± 0.1	40.93 ± 0.23	39.59 ± 0.05	0.54 ± 0.14	0.86 ± 0.54	na
Co (/ppm)	2.00 ± 0.05	0.50 ± 0.08	1.30 ± 0.12	nt^b^	nt	0.50 ± 0.04	0.50 ± 0.03	nt
Cu (/ppm)	8.50 ± 1.21	8.50 ± 0.58	21.50 ± 0.78	2.30 ± 0.16	3.80 ± 0.27	9.00 ± 0.65	16.00 ± 0.58	0.52
Fe (/ppm)	363.80 ± 4.35	31.75 ± 1.83	172.80 ± 8.14	23.80 ± 1.56	67.30 ± 1.58	71.00 ± 2.54	75.50 ± 2.69	0.18 ± 0.16
Mn (/ppm)	500 ± 1.78	16.75 ± 0.78	90.30 ± 1.53	5.30 ± 0.24	19.80 ± 0.47	40.00 ± 0.88	25.80 ± 1.24	22.46 ± 0.26
Mo (/ppm)	1.30 ± 0.54	3.00 ± 0.27	0.80 ± 0.31	3.30 ± 0.05	0.50 ± 0.02	7.25 ± 0.12	0.30 ± 0.23	1.21 ± 0.07
Zn (/ppm)	79.50 ± 1.26	47.00 ± 1.04	63.30 ± 0.85	48.50 ± 1.11	39.30 ± 0.06	73.00 ± 1.23	57.30 ± 2.39	56.71 ± 4.05
Se (/ppb)	168.80 ± 1.25	8884.30 ± 18.57	341.30 ± 12.21	611.30 ± 18.56	1371.30 ± 12.62	451.25 ± 11.54	516.30 ± 12.67	nt

a *Each value is an average of three replicates and is represented as mean ± standard deviation*.

b *Concentration lower than the detection limit*.

C *No analysis*.

The anaerobic sludge was collected from a pig farm in Changzhou Jiangsu, China. This sludge was kept in an anaerobic digester and fed daily with 1.5 g/L of glucose at the mesophilic condition for 1 month (Xi et al., 2014). After the feeding of glucose was stopped, the researchers waited until there was no more biogas produced by the sludge. Then, the seed sludge was removed from the digester, thoroughly mixed, and sieved through a 20 mesh filter screen. This was done to ensure the removal of easily degradable organic matter that were still present in the inoculum and to remove the dissolved methane.

### Batch AD Tests

Laboratory scale of 250 ml batch anaerobic digesters were constructed in the lab with 175 ml of working volume. Each digester contained two ports; one port for sludge sample withdrawal to analyze the process parameters like pH, COD, and VFA analysis during anaerobic digestion while the second port was equipped with a small evacuated bottle to collect the biogas samples for GC analysis. The experiments were carried out under mesophilic conditions. The HPR/wheat straw ratios (based on the TS of the wheat straw) were designed as 3, 5, and 10%, respectively. The C/N ratio was maintained at 30:1 by the addition of carbamide to the reactors. After the feedstock was added to the reactors, they were sealed immediately with butyl rubber stoppers, and the batch assay methane fermentation reactors were carefully checked for leakage and flushed with pure nitrogen (99.99%) for 3 min to ensure anaerobic conditions (Xi et al., 2015). Batch experiments were conducted in triplicate to determine the biogas production rates of wheat straw for 30 d. During anaerobic digestion, biogas samples were collected daily, and liquid samples were measured from the control digester in 3-day intervals for process stability investigation.

### Analytical Methods

Gas volume was corrected to the standard pressure (1.013^*^10^5^ Pa) and room temperature (20°C). The daily biogas production was obtained directly from the volume displaced, saturated NaHCO_3_ solution in the graduated cylinder after the mixture was manually stirred. The methane concentration in the biogas was analyzed using a gas chromatograph (GC 9890A, Renhua, China) equipped with a TCD (thermal conductivity detector), a TDC-01 column (4 mm^*^1 m, Shimadzu, Japan), and hydrogen as the carrier gas. The injector oven and detector temperatures were 100, 150, and 120°C, respectively. The flow rate of the carrier gas was 50 ml/min, and the sample injection volume was 0.5 ml.

The total solids (TS) and volatile solids (VS) were measured by the standard methods of the APHA (1998). The total carbon (TC) and total nitrogen (TN) contents were analyzed by a CHN (carbon, hydrogen, nitrogen) analyzer Vario EL (Perkin Elmer, USA). The pH was directly measured from the liquid samples with a digital pH meter (FE20K, Metter-Toledo, Switzerland). For the determination of the major and trace metal element contents, dried samples were pretreated with a mixture of HNO_3_/H_2_O_2_/HF, followed by neutralization with H_3_BO_3_, and the resulting clear solution was analyzed by the inductively-coupled plasma atomic spectrometry (ICP-OES, Thermo Fisher CAP 6200), according to the standard procedures. The contents of cellulose, hemicellulose, and lignin were determined by a sequential fiber analysis using the Goehring and Van Soest's method with a FIWE Cellulose Analyzer (Velp Scientifica Company, Italy) (Van Soest et al., 1991). Iodine-starch-spectrophotometric was used to detect the contents of starch in the materials (Li et al., 2012).

### Statistical Analysis

All analytical results were conducted at least in triplicate. The values of the different parameters were expressed as the mean and standard deviation. The standard deviations were analyzed using the Microsoft Excel 2003 for Windows.

## Results and Discussion

### Cumulative Production of Biogas and Methane Is Affected by the Addition of Different Ratios of HPR

Table 2 illustrates the cumulative production of biogas and methane when wheat straw was treated with different ratios of HPR additives compared with the control. When 10% Pg was added in the digester, the highest biogas production was achieved, which increased 28% in comparison to the control. In the same conditions, the highest methane production was achieved, which increased 37% in comparison to the control. These results agree with Xi who states that the best performance was achieved when 5% HPR was added to the reactor. Cumulative methane production increased by 31.4% compared to the mono-digestion of wheat straw (Xi et al., 2014). In some special cases, however, adding HPR also has negative effects. The gas productivity of the digester was inhibited when 5 and 10% of Am were added. It is worth noting that HPR has no effect on the biogas productivity in some of the experimental groups. There is no significant difference in the cumulative production of biogas and methane when treated with 3% of Ds compared to the control. As for the methane content, there is no big gap among the groups. The highest methane content was achieved when it was treated with 3% Ir residues, which reached 54.33%.

**Table 2:** Cumulative production of biogas and methane is affected by the addition of different ratios of HPR.

**Group**	**Biogas**	**Methane**	**Methane content**
	**ml/g.Vs**	**ml/g.Vs**	**%**
Control	263.33 ± 3.12	130.10 ± 1.16	49.40 ± 0.91
a1	310.00 ± 3.14	160.57 ± 1.08	51.80 ± 0.82
a2	243.62 ± 2.98	118.10 ± 1.32	48.48 ± 0.99
a3	233.14 ± 2.58	117.62 ± 1.13	50.45 ± 1.02
b1	266.57 ± 2.01	132.76 ± 1.42	49.80 ± 0.83
b2	325.05 ± 3.01	159.62 ± 1.12	49.11 ± 0.67
b3	316.57 ± 3.24	165.24 ± 0.98	52.20 ± 0.73
c1	309.90 ± 2.67	168.38 ± 0.87	54.33 ± 0.78
c2	314.95 ± 2.87	166.48 ± 1.24	52.86 ± 0.69
c3	306.76 ± 3.47	161.24 ± 1.21	52.56 ± 0.95
d1	296.86 ± 1.99	151.52 ± 1.17	51.04 ± 0.92
d2	314.00 ± 2.35	160.38 ± 1.15	51.08 ± 0.83
d3	337.24 ± 2.76	178.00 ± 0.93	52.78 ± 0.46
e1	288.57 ± 2.16	152.10 ± 0.99	52.71 ± 0.56
e2	316.86 ± 3.17	167.52 ± 1.18	52.87 ± 0.65
e3	317.81 ± 2.86	166.95 ± 2.01	52.53 ± 0.54
f1	304.10 ± 2.57	160.57 ± 2.02	52.80 ± 0.65
f2	305.52 ± 2.35	160.67 ± 1.01	52.59 ± 0.75
f3	328.48 ± 3.43	177.24 ± 1.45	53.96 ± 0.49

*a, b, c, d, e, f: Am residues, Ds residues, Ir residues, Pg residues, Cp residues, As residues. 1, 2, and 3: the ratios of HPR /wheat straw are 3, 5, and 10%, respectively*.

### Methane and Biogas Production Potential at Different Am Residues/Wheat Straw Ratios

**Figure 1:**
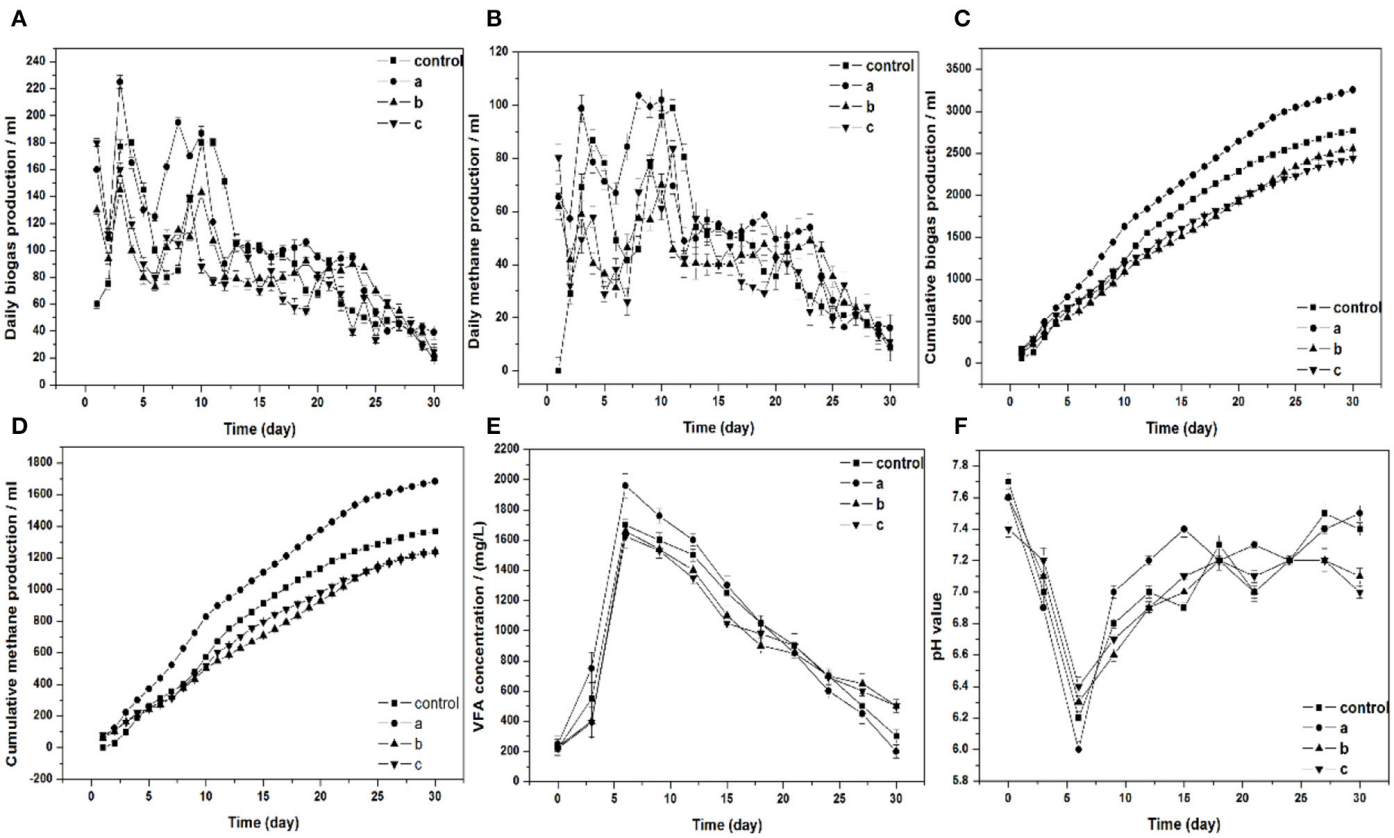
**(A)** Daily biogas production, **(B)** daily and cumulative methane production, **(C)** cumulative biogas production, **(D)** cumulative methane production, **(E)** VFA concentration, **(F)** pH value, respectively, in batch fermentation from wheat straw affected by adding different ratios of Am residues (a, b, and c: the ratios of Am residues/wheat straw are 3, 5, and 10%, respectively).

The effect of Am residues on biogas and methane production was illustrated in Figure 1. The highest cumulative biogas and methane production were achieved when treated with 3% Am residues, which yielded 3,541 ml and 1,869 ml, respectively. In comparison to the control, the biogas and methane production were 28 and 37% higher, respectively. Se was one of the main trace elements in Am. Formate dehydrogenase with Se, as a crucial component, oxidizes formate formation carbon dioxide to provide material for methane production (Fermoso et al., 2008). There have been many studies investigating that the supplementation of Se could provide gas production potential during the anaerobic digestion process (Facchin et al., 2013; Zhang et al., 2015). Furthermore, a negative effect occurred when 5 and 10% Am were added in the digesters, the production of biogas and methane were decreased compared to the control group. The group of 10% Am residues yielded the lowest biogas and methane production. These results suggested that the concentration of toxic chemicals reached the inhibition threshold when treated with 5 and 10% Am in the digesters. The daily biogas and methane production are shown in Figures 1A,B. The highest daily biogas and methane production were achieved with the addition of 3% Am which yielded 225 and 103 ml, respectively. Two obvious peaks were observed for the daily biogas and methane production in all of the digesters during their 30-d operation. The first peak of daily biogas and methane production appeared on the third day in the four experimental groups. Compared to the other experimental groups, the two peaks of control appeared at a much later period. As shown in Figure 1A, the daily biogas production in the group treated with 3, 5, and 10% Am residues reached 160, 130, and 180 ml, respectively, during the first 24 h of digestion. A temporary decline after the first day might be caused by the dissipation of substrate readily available for microbial decomposition (Ahn et al., 2010).

The concentration of volatile fatty acids (mainly acetic acid, propionic acid, and butyric acid) is the most important index in the anaerobic digestion process as it strongly affects the pH value and activity of methanogens (Zhu et al., 2010). VFAs formed is important in the anaerobic digestion process which can produce valuable end products. Acetic acid was produced by the degradation of propionate and butyrate through syntrophic acetogenic bacteria. Afterwards, it was decomposed into methane and carbon dioxide by acetoclastic methanogens (Montero et al., 2008). The irreversible acidification in the anaerobic digestion process is the major challenge for the anaerobic digestion which resulted in a rapid hydrolysis and acidogenesis, and it can cause the inhibition of methanogenesis or failure of the digestion (Veeken and Hamelers, 1999; Wang et al., 2009).

Figure 1E shows the concentration of VFAs during the 30 days of digestion. The highest VFAs concentration was achieved on day 6 for all groups. After 6 days, the VFAs concentration gradually declined. In comparison with the other groups, group a reached the highest VFAs concentration on day 6. Moreover, group a decreased to the lowest value at the end of digestion. It means that the highest concentration of VFAs was decomposed in group a during the process of digestion and production of biogas. In addition, the highest cumulative biogas production was achieved in a group a.

The pH is an important parameter for the adaptive operation and monitoring of the anaerobic digestion process. It strongly depends on the concentration of VFAs and buffering capacity of the fermentation broth because the synthesis of VFAs by acidogenic bacteria and pH value will decrease in the progress of fermentation. Figure 1F shows the variation of pH values during the 30 days of digestion. The lowest pH value in all groups occurred on day 6, which corresponds to the maximum VFAs production that was observed on day 6. Subsequently, pH values increased with a decrease in VFAs concentration, demonstrating the further conversion of VFAs to methane through methanogens. The final pH value in group a and the control were higher than that in groups b and c.

### Methane and Biogas Production Potential at Different Pg Residues/Wheat Straw Ratios

**Figure 2:**
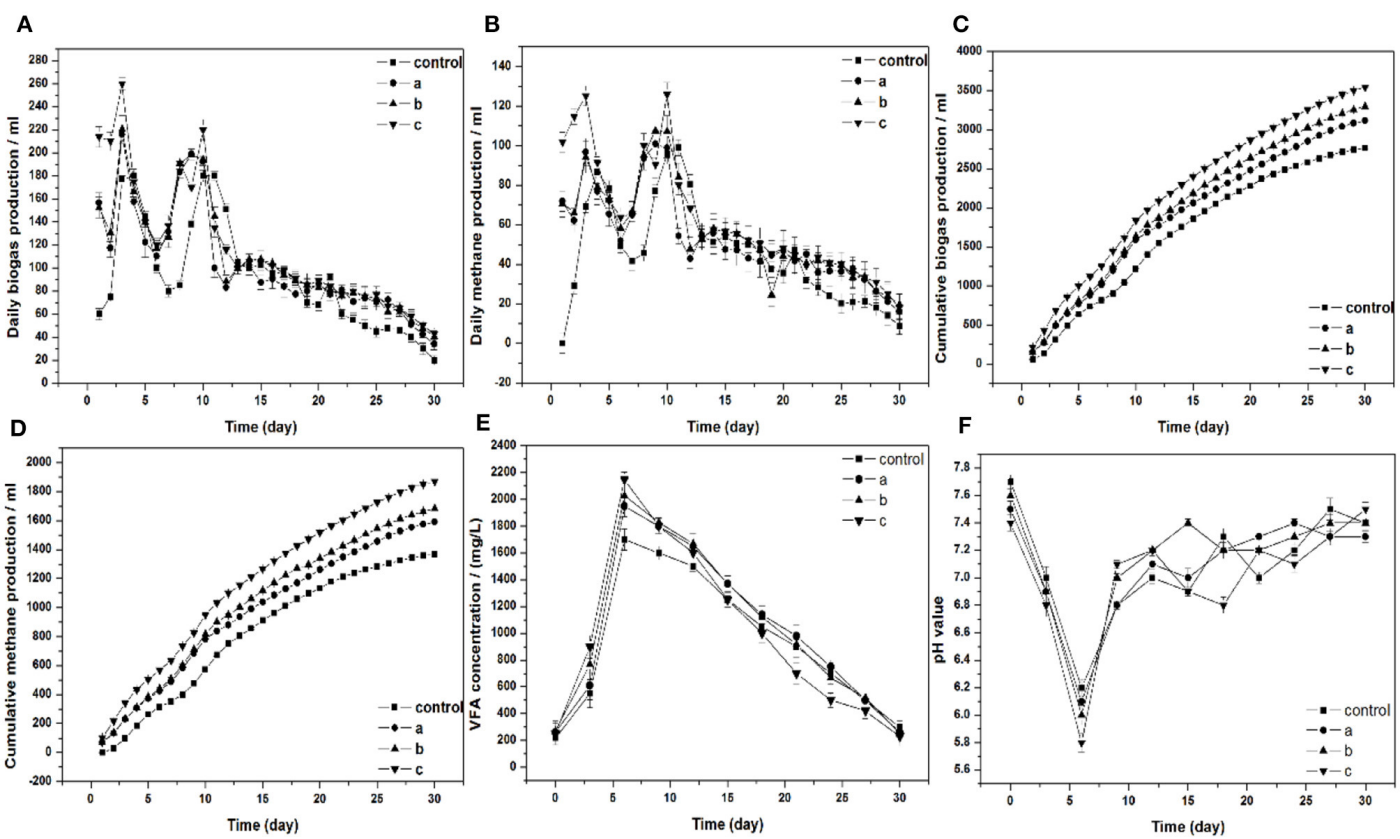
**(A)** Daily biogas production, **(B)** daily and cumulative methane production, **(C)** cumulative biogas production, **(D)** cumulative methane production, **(E)** VFA concentration, **(F)** pH value, respectively, in batch fermentation from wheat straw affected by adding different ratios of Pg residues (a, b, and c: the ratios of Pg residues/wheat straw are 3, 5, and 10%, respectively).

Figures 2A,B show the daily biogas and methane production. Two obvious peaks occurred in the four experimental groups. The daily biogas and methane production were the highest in group c, which yielded 260 and 126 ml, respectively. Compared to group c, the peaks of biogas and methane production increased by 44.4 and 28%, respectively. Cumulative biogas and methane were illustrated in Figures 2C,D. When Pg residues were added in the group, the cumulative biogas production and methane production obviously increased. The increased cumulative biogas production was 13, 19, and 28%, respectively. In addition, the cumulative methane production was 16, 23, and 37%, respectively. Pg residues are rich in the trace elements Se (Table 1). There have been many studies that investigated that the supplementation of Se could improve the biogas productivity in the digesters (Demire and Scherer, 2011; Bhatnagar et al., 2020). The highest cumulative biogas production and methane production were obtained in group c, which yielded 3,541 and 1,870 ml, respectively. In the group treated with 10% Pg, the cumulative biogas and methane production in the first 2 weeks accounted for 67% of the total production. Compared to the control, which accounts for 64 and 63%, respectively, both of them have no significant difference in the cumulative biogas and methane production of the first 2 weeks accounts for the total production.

Figure 2E displays the production of VFAs and variation during 30 days of the anaerobic digestion process. Because of the degradation of the organic components, the VFAs concentration increased significantly in the first 6 days of all groups. The VFAs concentration of all digesters reached the highest values of 1,700, 1,950, 2,028, and 2,148 mg/L (control, groups a, b, and c), respectively. The concentration of VFAs in groups a, b, and c were significantly higher than those in the control. In the next phase, the VFAs concentration gradually declined. The final VFAs concentrations of the control were 300 mg/L, which was higher than the other groups. These results correspond to the production of cumulative biogas and methane in all digesters. No methanogenesis inhibition has appeared in the case of the added Pg residues in the digesters, and the bacteria consumed VFAs quickly in the anaerobic digestion process.

Methanogens are highly pH sensitive and can work only in the narrow band of permissible pH of (6.5–7.8). Figure 2F displays the pH profile of the whole digestion process. The lowest pH value in all groups appeared on day 6, which was below 6.5. Subsequently, the pH value maintained stability in all the digesters. This illustrates that the anaerobic digestion system has a great buffering ability. The pH value was inversely proportional to the VFAs concentration shown in Figures 2E,F. As the VFAs concentration goes higher, the pH value started a declining behavior.

### Methane Production Potential at Different HPR/Wheat Straw Ratios

**Figure 3:**
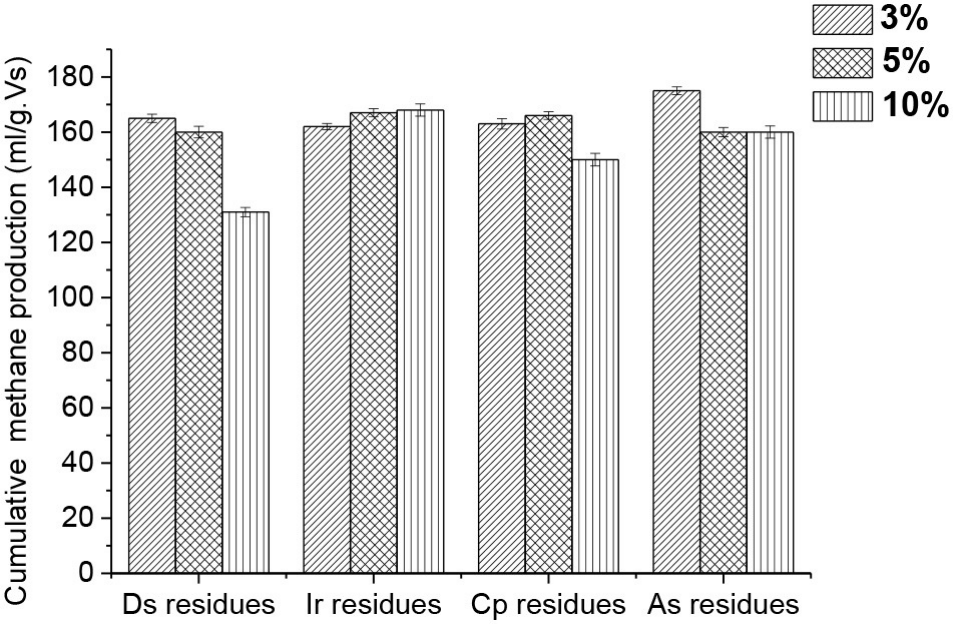
Cumulative methane production affected by the addition of different HPR in different ratios.

Figure 3 displays the cumulative methane production affected by the addition of different HPR in the value-added ratio. The cumulative methane production in all the groups is much higher than the control. Nevertheless, it is worth noting that the other groups did not suit the trend of the more HPR that was added, the more cumulative methane was produced, except for group b. This illustrates that the large amounts of trace elements in HPR have not been fully used up due to the bioavailable fraction which can be utilized during the whole anaerobic digestion process. Hence, the total contents of trace elements in HPR can no longer be a determining factor. There are many reactions between the solid and liquid phases in the digesters that were responsible for this phenomenon (Zandvoort et al., 2006). Such reactions are precipitation, co-precipitation, and adsorption, and so on. Sulfide chemistry is the main factor that causes these reactions, which had been claimed as the main regulator of the trace elements in the digesters (Fermoso et al., 2009). For instance, the metal sulfide of FeS is well-known to potentially and or co-precipitate Ni and Co, and then form the complexes of Fe-Ni/Co-S (Gustavsson et al., 2013; Shakeri Yekta et al., 2016).

### Perspective

1. The value-added ratio of HPR in the digesters has a significant effect on the production of biogas and methane.
2. The highest biogas productivity was achieved when treated with 10% Pg residues, which yielded 337 ml/g TS of biogas and 178 ml/g TS of methane. Cumulative production of biogas and methane increased by 28 and 37% compared to the 263 and 130 ml/g TS achieved in the control.
3. The production of biogas was decreased in the group treated with 5 and 10% Am residues.
4. Sometimes under the condition of adding the same HPR in different levels, the methane productivity was efficiently enhanced. Nevertheless, adding more HPR does not imply that the anaerobic digestion system can be run better with a much higher methane productivity.

## Data Availability Statement

The raw data supporting the conclusions of this article will be made available by the authors, without undue reservation.

## Author Contributions

XY and QX initiated the project and made suggestions, and revised the article. YX, YL, JD, and XK searched the database, wrote, and finalized the manuscript. All authors reviewed and commented on the entire manuscript.

## Conflict of Interest

The authors declare that the research was conducted in the absence of any commercial or financial relationships that could be construed as a potential conflict of interest.
